# Fate of bacterial indicators and *Salmonella* in biofilm developed on ultrafiltration membranes treating secondary effluents of domestic wastewater

**DOI:** 10.1038/s41598-018-36406-z

**Published:** 2018-12-24

**Authors:** Jeries Jadoun, Raghda Mreny, Ons Saad, Hassan Azaizeh

**Affiliations:** 1 The Galilee Society Institute of Applied Research, Shefa-Amr, 20200 Israel; 2 Department of Environmental Science, Tel Hai College, Upper Galilee, 12208 Israel

## Abstract

The fate of representative indicator and pathogenic bacteria on ultrafiltration (UF)-membrane surfaces treating secondary wastewater effluent, as well as their reaction to common biofouling-removal techniques was investigated. Field-condition experiments showed that the number of heterotrophic bacteria, fecal coliforms, *E. coli* and *Salmonella* on membrane surface increased rapidly and continuously until the end of the experiment, reaching 9, 6.5, 6, and 2.4 logs, respectively. Similar results were obtained under controlled laboratory conditions. However, the increase in the bacterial numbers was dependent on the supply of fresh wastewater. Quantitative real-time PCR verified the behavior of attached *E. coli* cells, although the numbers were 1–2 logs higher compared to the standard culture-based method. The number of attached bacteria was positively correlated to increases in DNA and protein content and negatively correlated to the membrane flux. *In-situ* membrane cleaning using sodium hypochlorite significantly reduced the number of attached bacteria. However, the effect was temporary and affected bacterial cell cultivability rather than viability. Taken together, these findings suggest that, under the studied conditions, indicator and pathogenic bacteria can initiate rapid biofilm development, persist on UF membrane surfaces, and survive membrane cleaning with sodium hypochlorite.

## Introduction

Water scarcity has become a global problem in arid and semiarid regions. The increasing demand for clean water has prompted the reuse of domestic wastewater and use of seawater as alterative water resources. Consequently, the conventional reclamation and desalination plants now face the challenge of operating at high performance and efficiencies to generate water of high quality and health safety standards^1^. Integration of membrane-filtration techniques in water treatment has become an attractive and popular solution to meet this challenge^1–3^. Among these techniques, ultrafiltration (UF) is considered the preferred technology for reclaiming wastewater and for pretreatment of wastewater and seawater prior to the reverse-osmosis (RO) stage^4^; this is because the UF process reduces colloidal, organic, and biofouling on RO membranes^5,6^. In wastewater treatment, UF can also be used during (in membrane bioreactor systems) or after (effluent polishing) the biological treatment^7–10^. Regardless of the treatment phase, UF membranes are themselves prone to severe fouling problems, particularly biofouling and organic fouling^2,11,12^.

Biofouling, which is a biofilm phenomenon, is a multistage process initiated by membrane conditioning via adsorption of macromolecules originally existing in the feed, such as proteins, humic acids, and polysaccharides, and the secretion of extracellular polymeric substances (EPS) by the microorganisms. Such membrane conditions facilitate attachment of one or more bacterial species to the membrane surface, followed by their growth and multiplication while utilizing the feed water nutrients, resulting in biofilm formation^2,12,13^. Biofouling (microorganisms with their EPS) is one of the most serious operational problems of membrane-based treatment systems and it is more complicated than other fouling forms due to the microorganisms’ ability to grow, multiply and spread to unoccupied sites on the membrane surface. Biofouling has several adverse effects. These include pore blocking and reduction in membrane water flux, an increase in solute concentration polarization accompanied by lower solute rejection, an increase in the module’s differential pressure, biodegradation and/or biodeterioration of the membrane polymer or other module construction materials, and increased energy requirements^12^. In addition, it has been suggested that biofouling also promotes the establishment of concentrated populations of human pathogens on membrane surfaces^13^. Such pathogens may also be a source for contamination of wastewater effluent downstream of the UF step. The various physical and chemical methods applied to control fouling and biofouling achieve only partial and temporary removal^12,14,15^. Given the potential public health risk of pathogenic bacteria accumulated on, attached to or penetrating the membrane, monitoring the fate of pathogenic bacteria on the membrane surfaces is very important.

Most analyses of the bacterial communities responsible for UF membrane biofilm/biofouling in wastewater-treatment systems have focused on either enhancement of treatment-process efficiency or reduction/prevention of biofouling^16–18^. In contrast, the fate of bacteria in the biofilm on UF membranes has received very little attention. Monitoring of pathogenic bacteria on filtration membranes is a challenge due to their originally low abundance in wastewater^19,20^, and presumably on filtration membranes^21^.

Quantitative real-time PCR (qrt-PCR) is a widely used microbial analytical tool in environmental biology and is considered a reliable substitute for the culture-based methods to quantify or monitor a specific microbial population, such as total coliforms and *Escherichia coli* in water^22,23^. Besides its high specificity and sensitivity resulting from targeting group- or species-specific genes, qrt-PCR also has the advantage of detecting bacteria in the VBNC state^24^. Hence, the objectives of this study were to investigate whether these bacteria can persist on the UF membrane surface following UF as part of the formed biofilm and how these bacteria react to techniques (sodium hypochlorite treatment) designed to control biofilm development.

## Results and Discussion

### Bacterial counts in wastewater effluents pre- and post-UF in the original UF unit of the RO system

The abundance of fecal coliforms (FC), *E. coli* and *Salmonella* sp. in the source wastewater and after UF treatment were determined using standard culture-based methods. As shown in Table 1, the reservoir pond, the UF feed and the reject stream all continued significant counts of the different bacteria. Expectedly, the UF completely removed *Salmonella* and resulted in 2.4 and 2.59 log removal of FC and *E. coli*, respectively. The occurrence of a few bacteria in the permeate was probably due to contamination. Notably, the numbers of bacteria in the UF reject (which includes the backwash permeate) were less than those of the UF feed suggesting that bacterial cells were still attached to the membrane surface. It is accepted that backwash is limited in its ability to remove complex forms of fouling, such as biofouling. Hence, to increase its effectiveness, chemical agents are added to the permeate or the backwash water^25^.

**Table 1:** *In-situ* bacterial counts^*^ pre- and post-UF determined via standard culture-based methods.

Sample source	Fecal coliforms (CFU/100 mL)	*E. coli* (CFU/100 mL)	*Salmonella* (MPN/100 mL)
Reservoir pond	8.88 × 10^4^	1.62 × 10^4^	40
UF feed	1.98 × 10^4^	8.25 × 10^3^	80
UF backwash + reject	1.65 × 10^4^	4.75 × 10^3^	20
UF permeate	77	21	0

^*^Numbers are averages of 4–5 separate sampling events.

### Monitoring of membrane-associated bacteria under field and laboratory conditions

**Figure 1:**
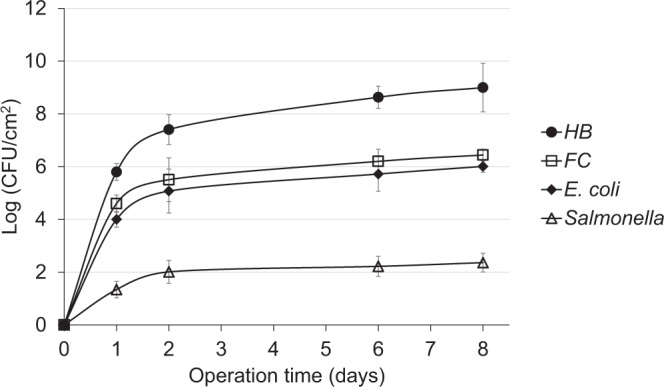
Counts (average of both membrane ends) of cultivable bacteria developed on the surface of UF membranes under field conditions. Data points are means of both ends of the two membranes ± standard error.

To gain insight into the fate of bacteria on the UF membrane surface, heterotrophic bacteria (HB), FC, *E. coli* and *Salmonella* were monitored on the membrane surface of the cross-flow cell systems under field, as well as controlled laboratory conditions. Microbial analysis of the biofilm samples collected from the RO-integrated flow cell system revealed a sharp and significant increase in the numbers of HB, FC, *E. coli* and *Salmonella* within 2 days; they continued to increase to 9, 6.5, 6, and 2.4 logs, respectively, by the end of the experiment (Fig. 1). The obtained HB counts were of the same order of magnitude as reported for nanofiltration (NF) membranes^26,27^. Interestingly, the ratio of the numerical increase of all bacteria, including *E. coli* and *Salmonella*, remained the same. This suggests that the existing *in-situ* UF conditions offer no selective advantage to either of these latter two bacteria. Overall, the continuous increase in FC, *E. coli* and *Salmonella* on the membrane surface despite washing indicates that indicator and pathogenic bacteria can successfully compete with other microbes present in wastewater and persist on UF membrane surfaces.

**Figure 2:**
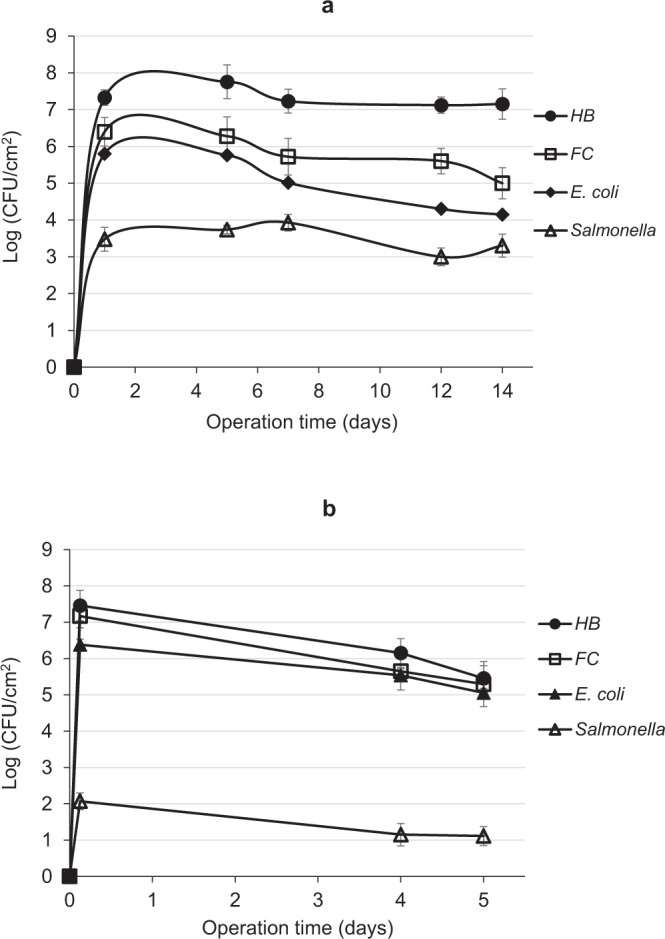
Counts of cultivable bacteria developed on the surface of UF membranes using the laboratory-scale system (1.5–2 bar, initial permeate flux 2.7–3.9 L/day) at (**a**) 22–25 °C (initial permeate flux 2.7 L/day); (**b**) 28–31 °C (initial permeate flux 3.9 L/day). Data points are means of three replicates ± standard error.

The bacterial counts in the biofilm developed on the UF membrane surface under controlled laboratory conditions were slightly different from those obtained for the RO-integrated UF system. In a temperature range of 22–25 °C, the numbers of HB, FC, *E. coli* and *Salmonella* increased to their maximum level and then remained constant for the first 5–7 days of operation, but then decreased (ending in ~1–2 log reduction) (Fig. 2a). This behavior was more pronounced when the experiment was repeated in a higher temperature range, 28–31 °C, where the numbers of the different bacteria began to drop continuously from the first day, eventually resulting in a 1–3 log reduction (Fig. 2b). Moreover, membrane clogging was more rapid. The decrease in bacterial numbers on the membrane surface correlated with that in the feed tank (data not shown), indicating that it very likely resulted from bacterial death due to the use of 4 °C-stored rather than fresh wastewater. Nevertheless, loss of cultivability due to the VBNC state might also be responsible. A recent study demonstrated that exposure to a low temperature of 4 °C under starvation results in a 3-log decrease of viable *E. coli* O157:H7 cells after 10 days of exposure^28^.

Interestingly, in both the *in situ* and controlled lab experiments, all the examined bacteria attached and accumulated on the UF membrane surface within 3–24 h of operation. Other studies also demonstrated the ability of various bacteria to attach and form biofilm within minutes and a few hours, respectively, on microfiltration and RO membrane as well as other surfaces^29–31^.

### Monitoring of membrane-associated bacteria in flow-cell system fed with wastewater and synthetic effluent mix

**Figure 3:**
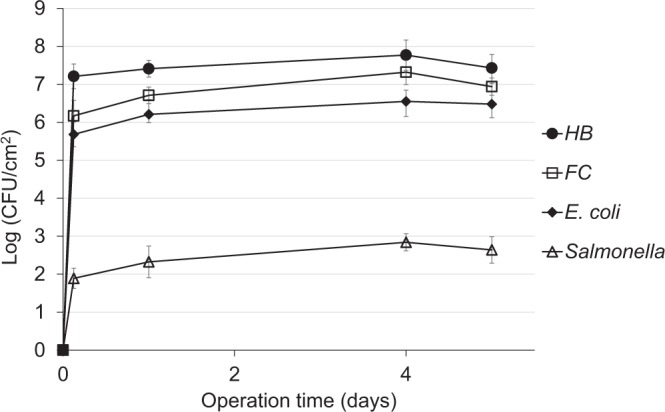
Counts of culturable bacteria developed on the surface of UF membranes using a laboratory-scale system fed with a 1:2 ratio of secondary to synthetic effluents (1.5–2 bar, initial permeate flux 2.7 L/day, 28–31 °C). Data points are means of three replicates ± standard error.

To prevent the death of bacteria in the feed, minimize stress due to unfavorable conditions, and to better assess the effect of temperature on bacterial accumulation on the membrane surface, the secondary wastewater effluent was mixed prior to feeding with synthetic effluent containing glucose as a carbon source^27^. Of the various ratios tested, the best secondary-to-synthetic effluent ratio that maintained a steady bacterial (HB) number (3 × 10^5^–3.16 × 10^6^ cfu/ml, or 5.5–6.5 Logs) was 1:2 (v/v) added to the starting mixture at 2- to 3-day intervals (data not shown). Under these conditions (1:2 mix, 28–31 °C), the bacterial behavior on the membrane surface in terms of quantity was very similar to that obtained with the RO-integrated flow-cell system, except for a slight decrease that was eventually observed toward the end of the operation (Fig. 3). Thus, indicator and pathogenic bacteria stably accumulate on UF membrane treating secondary effluents at ambient temperatures.

Bacteria accumulate on filtration membranes by two processes: attachment (bioadhesion and bioadsorption) and growth (multiplication)^27^. The correlation between the bacterial numbers present on the membrane surface and those in the wastewater feed suggests that under the conditions studied attachment was the main process contributing to the population increase on the membrane surface.

### Monitoring of *E. coli* and *Salmonella* on UF membrane surface using qrt-PCR

To confirm the culture-based quantification results, *E. coli* and *Salmonella* on the membrane surface were quantified by qrt-PCR, targeting the *lacZ* and *invA* genes, respectively. Although qrt-PCR showed high sensitivity for both genes, it failed to amplify the *invA* gene from DNA extracted from the biofilm samples. This could be due to the very low number of *Salmonella* accumulated on the membrane surface compared to *E. coli*, as observed using the culture-based methods (Figs 1 and 2).

**Figure 4:**
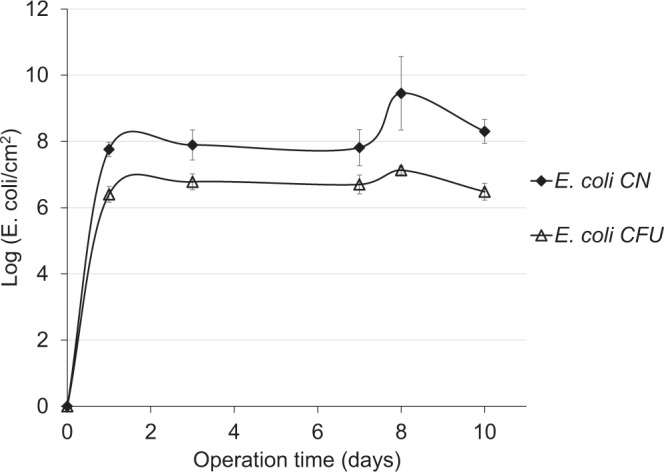
UF-membrane-associated *E. coli* counts using culture-based and qrt-PCR methods for the laboratory-scale system fed with a 1:2 ratio of secondary to synthetic effluents (1.5–2 bar, initial permeate flux 2.7 L/day, 28–31 °C). CFU, colony-forming unit. CN, gene copy number. Data points are means of three replicates ± standard error.

A comparison of culture-based methods and qrt-PCR revealed a positive correlation between the two methods for the *E. coli* numbers in both the integrated system (data not shown) and the laboratory-controlled flow-cell system (Fig. 4). However, whereas for early biofilm development, i.e., during the first hours of operation, comparable bacterial numbers were obtained for the two methods, the numbers obtained with qrt-PCR were 1–2 log higher than those obtained using culture media (Fig. 4). Higher qrt-PCR counts compared to plate counts have been reported in other studies^32–34^ and were attributed to the higher sensitivity of the qrt-PCR for quantification of bacteria in the VBNC state^33–35^. Higher qrt-PCR counts may also result from overestimation of the *E. coli* numbers on the membrane surface. Such an overestimation could result from the presence of free extracellular DNA and/or DNA derived from dead cells, due to the method’s inability to discriminate between live and dead microbial cells. This drawback can be overcome by treatment of the sample with ethidium monoazide and propidium monoazide prior to DNA extraction^36,37^. These compounds selectively enter bacteria with damaged cell membranes (i.e., dead bacteria) and bind covalently to the DNA after photoactivation, thus preventing PCR amplification of those cells^36,37^. In our study, such a treatment was not necessary because the culture-based methods had already demonstrated the persistence of *E. coli* on the membrane surface both *in situ* and under laboratory conditions. Another drawback of qrt-PCR is its limited throughput capacity^38^. The diversity of pathogenic bacteria in wastewater is extremely high, and detection of the different species by targeting their specific genes would be time-consuming^38^. The advent of next generation sequencing (NGS) techniques has resolved this problem. NGS techniques target the hyperpervariable regions of the16S rRNA gene and produce massive sequencing data, thus allow more adequate assessment of the microbial diversity. However, unlike qrt-PCR, profiles of species composition generated from NGS- based amplicon sequencing are considered as being qualitative. Moreover, NGS identification of pathogens at the species level is not always possible. Consequently, NGS is mostly applied for screening for pathogens, while qrt-PCR for their quantification and monitoring^39^.

### Membrane performance and biofilm matrix analysis

**Figure 5:**
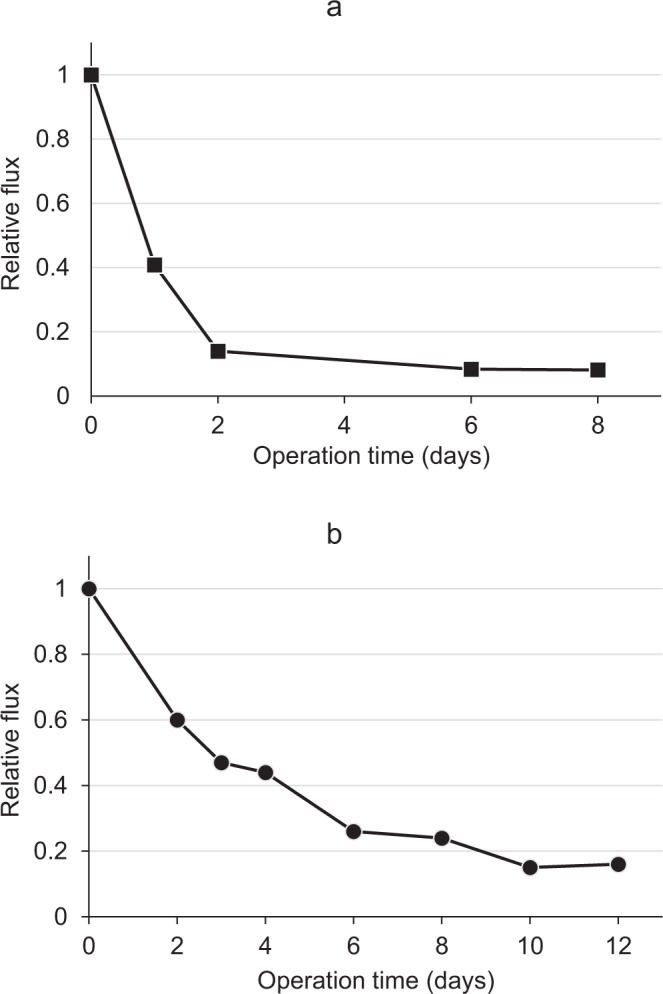
Relative flux of the UF membrane permeate. (**a**) Integrated flow-cell system under field conditions. (**b**) Laboratory flow-cell system (at 22–25 °C).

The flux profiles of the membranes as a function of time in both the integrated and laboratory flow-cell systems demonstrated a typical effect of bacterial accumulation on membrane performance (Fig. 5). The flux decline was very pronounced in the integrated flow-cell system compared to the laboratory system. Whereas in the former, a decline of 83% and 90% was observed after 2 days and 8 days of operation, respectively (Fig. 5a), in the latter, a decline of 76% and 82% was observed on days 8 and 10, respectively (Fig. 5b). Similar results have also been reported by Invitzky *et al*.^40^ using a cross-flow laboratory system, although a different membrane type (NF), feed wastewater (membrane bioreactor-treated domestic wastewater effluent), and operation conditions were used. As expected, the decline in the membrane flux was negatively correlated with the increase in bacterial numbers.

Bacteria in biofilm produce and release EPS that support and stabilize the biofilm structure^12^. EPS are mainly composed of polysaccharides, eDNA, proteins and lipids^41^. Consistent with their role, higher production of EPS occurs more extensively during the specific attachment stage of biofilm development^42^. To evaluate EPS production by the attached bacteria, total DNA and protein contents were extracted from the biofilm samples collected from the integrated and laboratory flow-cell systems and measured in parallel to the bacterial biomass and flux. The change in the content of both protein and DNA in biofilm membrane samples corresponded to that of the bacterial biomass, i.e., it increased rapidly, within hours, reached a maximum level and then decreased slightly. Thus, *E. coli* and *Salmonella* also contribute to the biofilm developed on UF membrane surface.

### Effect of chemical cleaning on biofilm-associated bacteria

**Figure 6:**
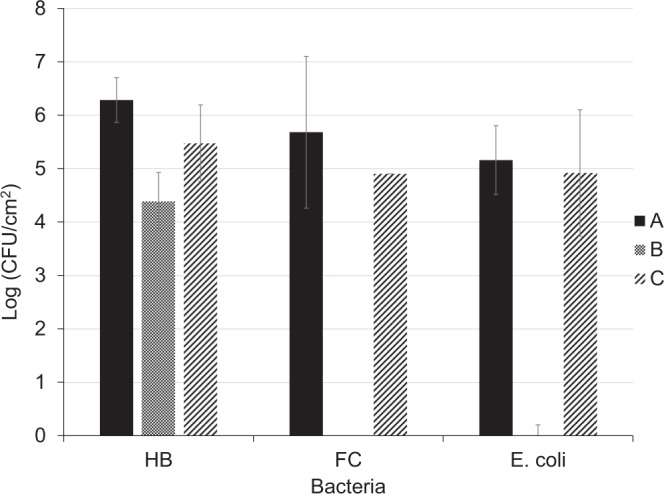
Bacterial counts of the different bacterial groups pre- and post-sodium hypochlorite treatment. A, Counts after 2–3 h of flow-cell system operation. B, Counts after treatment with sodium hypochlorite. C, Counts after an additional 2 h of operation with filter-sterilized feed. Data points are means of three replicates ± standard error.

Chemical cleaning agents can affect the microbial composition and density of the biofilm developed on membrane surfaces, resulting in selection for strongly adhesive microbial cells or EPS components, or both, particularly following repeated cleaning cycles^15,43^. Thus, the finding that *E. coli* and *Salmonella* were able to persist on the membrane surface prompted an investigation of the impact of treatment with sodium hypochlorite, one of the most commonly used chemical cleaning agents, on the viability of the attached *E. coli* and *Salmonella* compared to HB and FC. Treatment of the UF membrane for 0.5 h with 100 ppm sodium hypochlorite resulted in a 2.5 log reduction in the number of attached HB, and almost completely eliminated the attached FC and *E. coli* (5–5.5 log reduction). However, the effect was temporary as the numbers of all of these bacteria increased again within 2 h of renewed operation, to almost the same level as before the treatment (Fig. 6). Since in these experiments filter-sterilized secondary effluent was used for feeding the flow-cell system, it can be assumed that the observed rapid and significant increase in the bacterial population resulted from recovery of bacterial cultivability; i.e., sodium hypochlorite affected the cultivability rather than the viability of the attached bacteria. Piasecka *et al*.^15^ showed that although treatment with sodium hypochlorite at successive concentrations of 40–400 ppm significantly reduced the richness and density of bacteria attached to the PVDF membrane of a laboratory-scale membrane bioreactor system 1 day after treatment, a concentration of 4000 ppm was required to completely remove the bacteria from the membrane surface. Thus, better biofilm reduction could be achieved by membrane cleaning using higher sodium hypochlorite concentrations.

## Conclusions

It has been suggested that biofouling promotes the establishment of populations of water-borne pathogens on membrane surfaces treating wastewater. Bacterial monitoring experiments on UF membrane under field and controlled laboratory UF conditions using both culture-based and qrt-PCR indeed demonstrated the ability of bacteria, such as *E. coli* and *Salmonella*, to establish rapid biofilm formation and persist on the membrane surface. This conclusion was further supported by the correlation between bacterial persistence and decline in membrane flux associated with an increase in protein and DNA contents in membrane biofilm. In terms of methodology, although qrt-PCR resulted in higher bacterial counts, both the culture-based and qrt-PCR methods are adequate for monitoring of bacteria on membrane surfaces applied to the treatment of municipal secondary wastewater effluents. The survival of biofilm-forming bacteria on the membrane surface despite *in situ* backwash and treatment with sodium hypochlorite highlights the importance of optimization of the treatment process for efficient control of biofouling. Taken together, the persistence of indicator and pathogenic bacteria, such as *E. coli* and *Salmonella*, respectively, on UF membranes and their incomplete removal by common physical and chemical membrane cleaning methods are important issues that should be considered for safer membrane use and disposal.

## Methods

### Pilot-scale UF pretreatment unit, UF flow-cell systems, sampling and sample preparation

**Figure 7:**
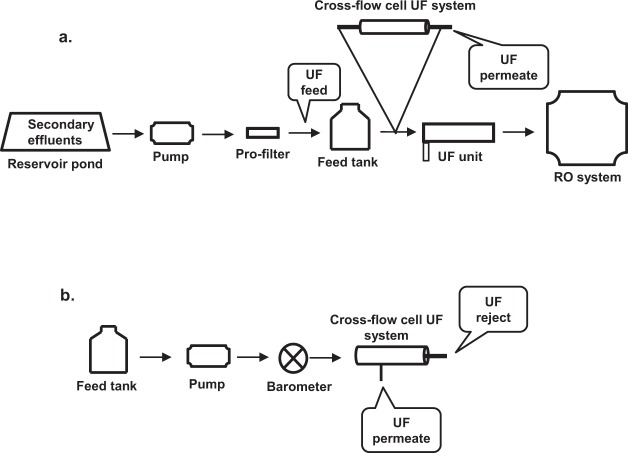
Schematic diagram of the RO- integrated (**a**) and the laboratory cross-flow cell (**b**) systems.

The UF unit was part of a pilot-scale RO system used to treat municipal secondary (activated sludge-treated) effluent. Secondary effluent collected in a reservoir pond was filtred through a 50-µM membrane filter and continuously pumped into the UF unit. The UF membrane was backwashed three times per hour to prevent fouling. Wastewater samples (0.5–2 L) from the UF system were collected during a period of approximately 1 week along the various pretreatment train (reservoir pond, UF feed, UF reject stream which also included the backwash, and the permeate) prior to the RO system treating secondary municipal effluent of an activated sludge system (Fig. 7a).

The two laboratory-scale UF cross-flow cell systems used in this study included tubular polyvinylidene fluoride (PVDF) UF membranes (cutoff of 100,000 MW; FP100, PCI membrane, Xylem Inc., USA) with the same properties as the membrane of the UF unit of the pilot scale reverse osmosis (RO) system. The first UF cross-flow cell system was connected to the RO system in parallel to original RO-UF unit and it consisted of two membranes, each 20 cm in length, that were serially connected in a stainless-steel tube (Fig. 7a). The second system consisted of one 10-cm long UF membrane, and used for laboratory experiments (Fig. 7b).

Wastewater secondary effluents collected from the reservoir pond, alone or enriched with synthetic effluent, were pumped into the system under two different temperature ranges, 22–25 °C and 28–31 °C, and at 1.5 bar, resulting in an initial permeate flux of 2.7–3.9 L/day. When mentioned, the synthetic effluent contained NaCl, 300 mg/mL; CaCl_2_, 50 mg/mL; MgSO_4_, 50 mg/mL; NH_4_Cl, 1.4 mg/mL; H_2_NaPO_4_, 0.29 mg/mL and glucose to meet a theoretical chemical oxygen demand concentration (COD) of 20 mg/L at a COD:N:P ratio of 100:5:1, which is known to be optimal for bacterial growth^44^.

Biofilm samples were collected from the membrane surface of the integrated and laboratory-scale flow-cell systems at different operation-time intervals (until complete clogging of the membrane) as described by Ivnitsky *et al*.^40^. Briefly, after temporarily stopping the flow and opening the flow cell or external UF module, the membranes were washed three times to remove unattached or loosely attached fouling layers. A 1 cm^2^ area of the surfaces at both ends of the membranes were swabbed, each time in a different place. Swabs were then placed in 0.5 mL of phosphate-buffered saline (PBS)–Tween solution [PBS + 0.05% (v/v) Tween 20] and were vortexed to release the attached biofilm. Aliquots (100 μL) were then either immediately analyzed for quantification of bacteria or stored at −20 °C for later determination of protein and genomic DNA content, as well as for bacterial quantification using qrt-PCR.

### Direct microbial analyses using conventional methods

HB and the commonly used fecal indicators FC and *E. coli* (also used as an indicator for pathogenic bacteria), as well as the pathogen *Salmonella* sp. in biofilm samples were enumerated according to standard methods, except for dilutions were performed in smaller volumes^45^. Briefly, HB (total) were enumerated by plating 10-fold serial dilutions of samples on R2A agar and incubating at 37 °C for 3 days. The FC were enumerated by the membrane-filtration method and plating on MFC agar followed by incubation at 45 °C. *E. coli* were enumerated by replica plating on nutrient agar supplemented with 4-methylumbelliferyl-beta-D-glucuronide (MUG) and counting fluorescent colonies after incubation at 37 °C for 4 h. *Salmonella* sp. were enumerated by the most probable number (MPN) method following enrichment in selenite broth at 37 °C and confirmation by growth on xylose lysine deoxycholate agar and then on triple sugar iron agar and lysine iron agar.

### Genomic DNA extraction and purification

DNA was extracted from biofilm samples using the method described by Lemarchand *et al*.^46^, with glass beads instead of ceramic beads and a higher volume of lysis buffer. Briefly, 1 mL extraction buffer (50 mM Tris–HCl pH 8.0, 5 mM EDTA, 3% w/v SDS, 10 μg/mL RNase A) and 1.5 g glass beads were added to the biofilm sample and cells were lysed by beating the mixture at room temperature at maximal speed using a bead beater. Following centrifugation, the supernatant was transferred to a new epi tube and impurities were precipitated by adding ammonium acetate (2 M final concentration), incubating on ice, and centrifugation. The supernatant was then extracted with 500 μL phenol/chloroform/isoamyl alcohol (25:24:1, v/v) solution and chloroform. Finally, the DNA was precipitated by centrifugation following the addition of an equal volume of isopropanol. The DNA pellet was rinsed twice with 70% ethanol and the pure DNA was dissolved in 50 μL ultrapure (DNase-free) water and stored at −20 °C for analysis. This method was also used to extract DNA from pure culture pellets of *E. coli* (ATCC 25922) and *Salmonella typhimurium* (ATCC 14028). Cell pellets of these bacteria were collected by centrifugation (5 min, 14,000 *g*, room temperature) of 1.5–3 mL bacterial culture grown to log phase in tryptic soy broth at 37 °C. The obtained A260/A280 and A260/A230 values were between 1.8 and 1.99 and above 1.5, respectively indicating that protein contamination was negligible and the DNA of good quality.

### Quantification of protein content in biofilm samples

Biofilm sample protein content was determined using the Bradford method. A sample aliquot (100 μL) was treated with 0.5 M NaOH and incubated for 45 min at 55 °C and an additional 15 min at room temperature. The extract (10 μL) was then added to 190 μL ultrapure water and 50 μL Bradford reagent (Bio-Rad). Protein content was assayed in a 96-well plate by a microplate reader at a wavelength of 595 nm. Bovine serum albumin (1 mg/mL) was used to prepare standard curves.

### TaqMan qrt-PCR

Real-time PCR was carried out using oligonucleotides and hydrolysis probes targeting *E. coli lacZ* and *Salmonella* sp. *invA* genes^47,48^. Both of these genes appear in one copy in the genome, and therefore copy number was equivalent to colony-forming units (CFU). Probes were labeled with 6-carboxyfluorescein (FAM) and quenched at the 3′ end with BHQ-1 (TAG Copenhagen A/S). The rt-PCR were prepared with ABsolute™ Blue qPCR ROX Mix (ABgene®, Thermo Scientific). Each reaction contained 1X PCR mix, 200 nM probe, 300 nM of each primer and 1–5 µL template (10 ng DNA, or serial 10-fold dilutions of this concentration for the calibration curves) in a 25-µL reaction volume. Reactions were carried out using the Eco qPCR system (Illumina) as described by Foulds *et al*.^47^ with the exception of increasing the annealing/extension from 60 s to 75 s. Gene copy numbers were determined using a calibration curve generated by plotting 10-fold serial dilutions of known concentration of *E. coli* or *Salmonella* DNA against the threshold cycle (Ct), and the gene copy numbers were determined as described^49^. Using the above conditions, high qrt-PCR efficiency was achieved for both *lacZ* and *invA* genes, detecting 20 gene copies each.

### Chemical cleaning of the UF membrane

The UF membrane of the laboratory-scale cross-flow system was cleaned *in situ* (without membrane removal), using an experimental setup similar to that described by Rabuni *et al*.^50^. Following 2–3 h of UF of fresh secondary effluent, the UF membrane was cleaned by replacing the secondary effluent feed with 100 ppm (in water) solution of sodium hypochlorite (NaOCl) and running the filtration for 0.5 h. This concentration falls within the range studied by other researchers^15^. The sodium hypochlorite solution was then replaced with filter- sterilized (0.45 μM) fresh secondary effluent. After another 2 h of UF, the filtration process was stopped and the membrane was sampled. Thus, samples were collected at three points: (i) after 2–3 h of operation of the flow-cell system before sodium hypochlorite treatment; (ii) after 0.5 h exposure to sodium hypochlorite; (iii) after an additional 2 h of operation (post-sodium hypochlorite exposure) with filter-sterilized secondary effluent. All samples were then subjected to microbial analysis (enumeration) as described above.

The datasets generated during and/or analysed during the current study are available from the corresponding author on reasonable request.
